# The use of text messaging for improving adherence to anti-diabetic regimen and glycaemic control in low-resource settings of South Africa: A study protocol for a randomised controlled trial

**DOI:** 10.1016/j.conctc.2019.100418

**Published:** 2019-07-19

**Authors:** Eyitayo Omolara Owolabi, Daniel Ter Goon

**Affiliations:** Department of Nursing Science, Faculty of Health Sciences, University of Fort Hare, East London, South Africa

**Keywords:** Diabetes, mHealth, Text messaging, South Africa, Low-resource settings

## Abstract

**Background:**

Despite the various innovations and treatment modalities, diabetes treatment outcomes remain sub-optimal globally and in South Africa. This places significant strain on the healthcare system. Text messaging is a cost-effective measure for promoting health. Yet, its effectiveness in improving diabetes treatment outcomes has not been documented in South Africa, especially in the resource-restrained settings. This study aims as determining the effectiveness, feasibility and acceptability of text messaging in improving diabetes outcomes in low-resource settings of the Eastern Cape Province of South Africa.

**Methods and analysis:**

This is a multi-centre, two-arm, parallel, randomised controlled trial which will compare the use of text messaging in addition to diabetes standard care to standard care alone in promoting diabetes outcomes for six months. The study will be conducted at six primary healthcare centres in two selected districts in Eastern Cape, South Africa. The study requires 216 participants if an attrition rate of 20% is anticipated. Data will be collected at baseline and six months. Randomisation will follow baseline data collection using simple randomisation, with an allocation ratio of 1:1, after removing any identifying information. The primary outcome measure is a change in mean morning random blood sugar. Secondary outcomes include change in diabetes knowledge, medication adherence, self-management, health-related quality of life, mean blood pressure, weight and clinic attendance. Data will be obtained through self-reporting using validated measures, review of clinical records and objective measurements. Data collection and measurements will follow standard procedure. Data analysis will be based on the principle of Intention-to-treat.

**Trial registration:**

This trial was registered in the Pan African Clinical Trial Registry, trial number: PACTR201810599931422.

## 1 Introduction

Diabetes, one of the four priority non-communicable diseases (NCDs) continues to constitute significant public health and socio-economic threat worldwide [1]. It is a leading cause of death and disability, with a continual increase in prevalence and associated mortality across several countries [2,3]. Against previous knowledge of being a disease found predominantly among the rich nations, developing nations are currently experiencing a swift increase in the burden of diabetes, especially among the poorer population [[4], [5], [6], [7]]. Worryingly, the burden of diabetes in under-resourced settings is complicated by a lack of effective and adequately-equipped healthcare systems and inadequate prevention strategies [8].

A surge in the prevalence of diabetes has been documented in South Africa with an increasing disease burden and associated mortality [9]. South Africa has the second highest prevalence of diabetes in Africa [10] and diabetes is the second leading cause of morbidity and mortality in South Africa [11]. Despite the effectiveness of drug therapy in diabetes management, high rate of poor adherence with therapeutic regimen persist among individuals with diabetes which contributes to poor glycaemic control [12,13].

Achieving glycaemic control among individuals with diabetes in South Africa appears to be a challenging task for healthcare professionals [14]. This is most likely due to documented poor adherence to medication and lifestyle therapy and poor knowledge among individuals with diabetes attending primary healthcare facilities, amidst other factors [15,16]. As a result, there is a resultant predisposition to complications development leading to a poor quality of life, disability, premature mortality and excessive burden on the individuals.

mHealth is an emerging and a cost-effective measure proven to be effective in improving patients’ self-management behaviours, knowledge, adherence to therapeutic regimen and compliance with appointments [[17], [18], [19]] though, sometimes, there are mixed results [20]. The effectiveness of mHealth among individuals with diabetes has rarely been documented in South Africa, and more specifically, in an economically poor region of the Eastern Cape with high rate of uncontrolled diabetes [21]. Hence, the need for an interventional study to determine the efficacy of mHealth technology, aside the usual care in promoting adherence to anti-diabetic therapy and glycaemic control among individuals with diabetes in a low-resource setting. The particular mHealth technology of interest is the use of mobile phone text messaging, which is considered a cost-effective measure of improving health outcomes [[22], [23], [24], [25]].

Mobile phone SMS is a documented measure for transmitting health information, even to the most remote areas of the world [26,27]. Also, the mobile phone SMS could be used to foster diabetes patients' knowledge and promote adherence to recommended therapy and improved treatment outcomes [25,28,29]. Considering the chronic nature of diabetes and the importance of patients’ involvement, improvement in self-management behaviour which could result from the SMS intervention will contribute to improved outcome and quality of life of the patients. The findings of this study will help to ascertain if the documented effectiveness in other countries and settings is feasible, acceptable and effective in resource-limited and remote areas as this setting. Likewise, the findings of the study could help inform policy makers to develop preventive strategies for diabetes and other chronic illnesses, using cost-effective and innovative technologies.

## 2 Research aim and objectives

The aim of this study is to determine the effectiveness, acceptability and feasibility of daily SMS in addition to standard care as a tool in promoting adherence to anti-diabetic regimen, glycaemic control and other clinical outcomes among diabetic individuals with poor glycaemic control.

### 2.1 The objectives of the study are

1. Determine the impact of daily SMS on glycaemic control, blood pressure and weight of diabetic patients.
2. Assess its impact on adherence to medication and diabetes self-management
3. Evaluate the effectiveness of the daily SMS as a clinic appointment reminder
4. Assess its effect on the health-related quality of life of the study participants.

### 2.2 Research hypotheses

The following hypotheses are framed for the study:

i. Participants receiving the daily SMS in addition to the standard care will show better improvement in diabetes knowledge and medication adherence compared to those receiving standard care alone.
ii. Patients receiving the daily SMS in addition to the standard care will show more improved clinical outcomes in terms of change in mean blood pressure and body weight compared to those not receiving the SMS.
iii. Diabetic participants receiving the daily SMS in addition to standard diabetes care will show better improvement in glycaemic control compared to those not receiving SMS.
iv Participants receiving the daily SMS in addition to the standard of care will have a better health-related quality of life compared to those receiving standard care alone.

## 3 Methods/design

This study protocol describes a multicentre, two-arm, parallel, randomised controlled trial aimed at assessing the effectiveness of mobile phone SMS intervention in addition to diabetes standard care in promoting adherence, glycaemic control measured as change in mean morning random sugar, and other clinical outcomes among diabetic patients for six months.

This study protocol follows the 2013 Standard Protocol Items: Recommendations for Interventional Trials statement [30] while the description of the intervention followed the CONSORT checklist [31].

## 4 Study setting

The study will be conducted at six selected primary healthcare centres out-patient departments in two districts in Eastern Cape, South Africa. There are no clear registers to ascertain the number of diabetic patients attending these clinics as the primary healthcare system record keeping is flawed in several ways. However, based on the prior interview done with the head of the facilities, approximately 15–20 diabetics are seen daily at the outpatient departments, making a total of about 180–300 diabetic patients per month, based on an average of three clinic days per week.

## 5 Study population

The study population are adults with uncontrolled diabetes attending the selected primary healthcare clinics at low-resource settings in Buffalo City Metropolitan Municipality and Amathole health districts, who meets the eligibility criteria.

The eligibility criteria includes being 18 years of age and above; diagnosed of diabetes at least in the last 6 months; currently receiving treatment at selected clinics; on a stable medication for at least three months prior to recruitment; a HBA1c level >7% or a morning random blood sugar more than 10 mmol/L; possession of a mobile phone with ability to retrieve and read SMS or have someone who is available to help with reading the SMS daily; willingness to receive SMS for the period of the study.

The exclusion criteria include having health or mental conditions that could interfere with the study or the ability to use the mobile phone or comprehend messages, pregnancy or plan to get pregnant in the next six months, debilitated or handicapped in such a way that obtaining anthropometric measurements could be challenging.

Eligible participants will be recruited sequentially at the selected health facilities on the clinic days. Recruitment process started in July–August 2018. Intervention commenced in September which is to run till end of February before the commencement of the post-intervention data collection.

## 6 Sample size calculation

The previously reported mean HBA1c in the setting was 10.6% and standard care for diabetes using metformin has been reported to reduce HbA1c by 1% (11 mmol/L) [32,33]. Assuming the intervention adds an extra 0.5% and considering a standard deviation of 1 and an alpha error level of 5%, the two-tailed calculation gives a power of over 90% with only 84 participants in each of the control and intervention group. If 20% loss of participants to follow-up is anticipated [24], a total of 108 participants is required in each group.

## 7 Randomisation

**Fig. 1:**
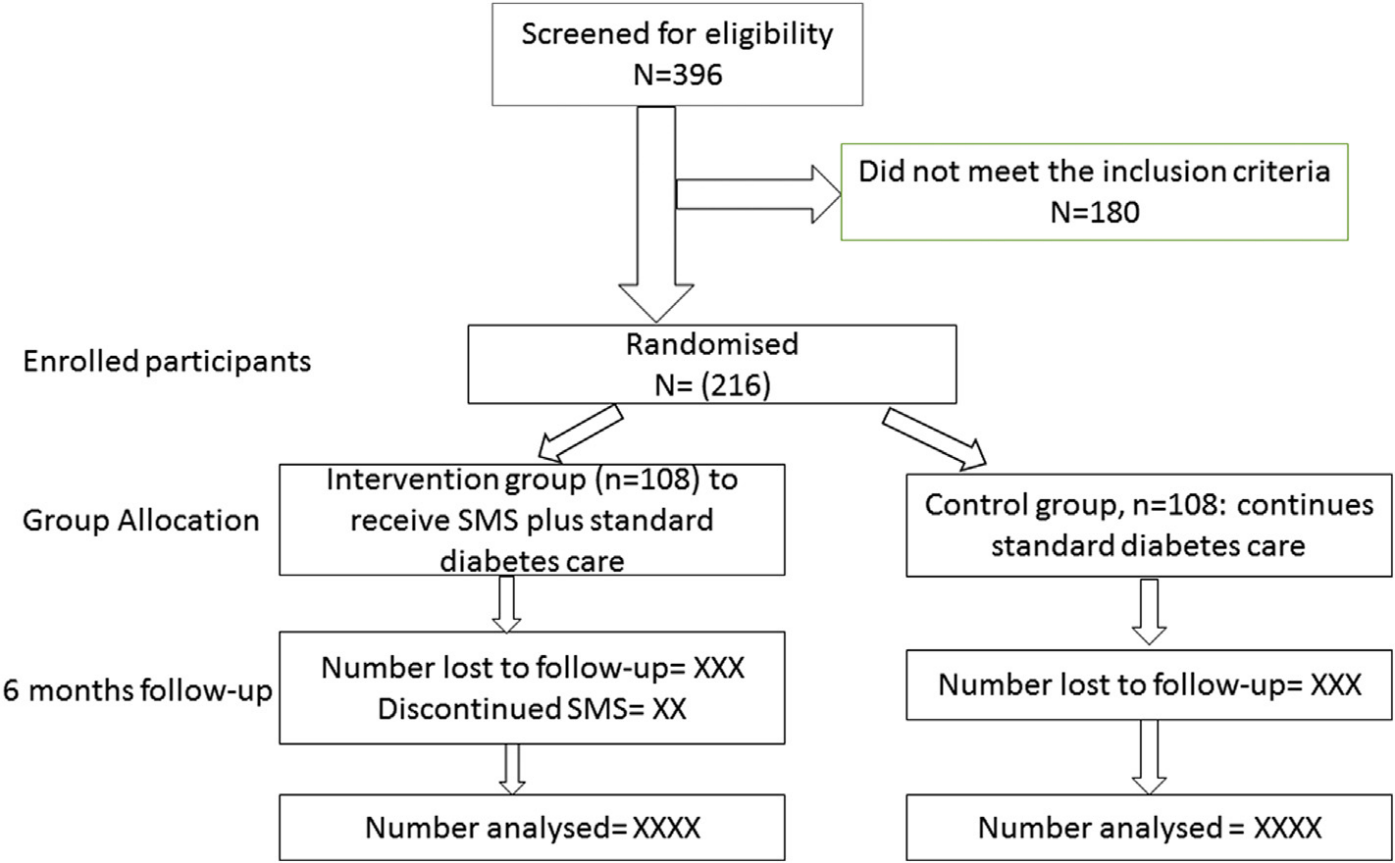
Flowchart for recruitment process.

The guideline for the management of diabetes is similar across all the primary health care facilities in South Africa [32], thus, all clinics are considered eligible; although the quality of care might vary across various health facilities as a result of available infrastructures as well as the available human resources and the experiences of the care providers. Of all the eight municipalities/districts, two were conveniently selected. In each selected district, the diabetes clinics were assessed for available facilities such as human resources, HbA1c testing, available diabetes support programmes. The information were obtained from the various facilities or unit heads as deemed appropriate. The obtained information were scored and used to stratify the diabetes clinics into two levels; average and low resourced clinics. All the clinics were assigned an identification number which were hidden from the study statistician. One average-resourced and two low-resourced clinics were selected from the two selected districts, summing up to 6 clinics; 4 low resourced and 2 high resourced clinics. From the clinics selected, demographic and other basic information were obtained to screen for eligibility. From the sample size calculation, 108 participants are required in both arms of the study, therefore, 36 participants are required from each of the six selected clinics. After the collection of the baseline data, randomisation will take place, where individuals are randomly assigned to either the intervention or the control arm using a simple randomisation technique with a 1:1 allocation ratio using the assigned ID numbers. The study statistician will randomly select 36 participants from the list of eligible participants from each clinic, adjusting for age and mean duration of diabetes. Participants in the intervention arm will then be contacted to ascertain their preferences in terms of the preferred language of communication; either the locally spoken Xhosa language or English, preferred time of receiving SMSs, name and contact of next of kin or the available support person (see Fig. 1).

## 8 Blinding

The study statistician involved in the randomisation will be blinded to every identifying information. Due to the nature of the study, it will be impossible for the research staff conducting the SMS intervention and the patients in the intervention arm to be blinded to the intervention. However, the primary outcome, the random blood sugar which is an objective measure, and other secondary outcomes will be blinded to treatment allocation.

## 9 SMS development

The contents of the SMS to be used for the study were developed by a team involving the principal investigator, supervisor, family physician, endocrinologist and a nurse. The team followed the Society for Endocrinology, Metabolism and Diabetes of South Africa (SEMDSA) guideline for the management of diabetes, the health education materials from the National Diabetes Education Programme and some sample messages from the previously documented pilot studies which were documented to be efficient [[18], [24]]. Also, the health care needs of the participants was not neglected. The SMS was developed in English and translated to Xhosa by a professional translator. The two versions of the messages were then pre-tested by sending them out to various people, including those with little or no level of education, to ascertain simplicity and clarity. Modifications were made using various feedbacks received.

## 10 Quality control and data management

In order to ensure accuracy of data, the same team constituting the principal investigator, and trained research assistants will conduct all interviews, measurements and data collection at all the centres. The team led by the PI will constantly check the data for completeness and accuracy with constant feedbacks from the team. The obtained data were captured on an excel spreadsheet, using double-entry measure to improve accuracy. Only the PI will have access to the data.

## 11 Outcome measures assessment

The assessment of the outcome measures will be conducted at baseline and at 6 months after randomisation. At baseline, demographic data, self-reported measures will be obtained using a validated questionnaire via face-to-face interviews, and clinical measures will be obtained from the available clinical records while the assessment of blood glucose level will be carried out following standard procedure.

The primary outcome measure is the change in mean morning random sugar. The choice of assessing the random blood glucose was made after preliminary investigations at the various primary healthcare clinics indicated that majority of these diabetic patients only get to check their HbA1c level once in a year. Similar report was documented by Daramola's [34] study conducted among diabetics in another province of South Africa. As such, relying on the HbA1c as a measure of making clinical judgement regarding glycaemic control may be unrealistic in this setting and most especially at this level of healthcare where a large number of these patients are being managed. Several studies have long correlated the fasting and random blood sugar with HbA1c [[35], [36], [37], [38]]. Although it is not acceptable as a gold standard for measuring glycaemic control, it could however be an alternative measure in low-resource settings as this, where obtaining HbA1c level still seems to be challenging.

Demographic information and clinical history will include age, gender, level of education, employment status, income, duration of illness, presence of other comorbidity and medication use.

Other secondary outcome measures will be obtained used previously validated tools:

• Knowledge of diabetes will be obtained with the validated Simplified Diabetes Knowledge Test Questionnaire-2, the true/false version [39].
• Medication adherence will be assessed with a self-designed, medication adherence questionnaire
• Health Related quality of life will be assessed with the EQ-5D quality of life questionnaire [40].
• Diabetes self-management behaviour using the Diabetes Self-management questionnaire [41,42].
• Co-morbid outcomes (hypertension and obesity) will be obtained through blood pressure measurement and anthropometric measurements (body weight, height, and waist and hip circumference).
• Compliance with appointment will be assessed via the appointment and visit records of the patient.
• Behavioural characteristics (smoking, alcohol use and physical activity using the WHO STEPwise approach.
• Acceptability of the SMS intervention using the participants' feedback obtained through some sample questions from a validated tool [18]
• Feasibility of the study was assessed by the recruitment and the retention rates

## 12 Study intervention procedure

Both the intervention and the control groups will proceed with their usual care including all medical visits, tests and diabetes support programmes. In addition, the intervention group will receive short message services (SMS) at an agreed time of the day, tailored according to their needs, care plan and goals. Participants will as well receive motivational and support messages, and advice on lifestyle behaviours like diets, physical activity and smoking cessation and medication and appointment reminders. Where appropriate to their care, participants will also receive reminders to check their blood glucose levels. Some samples of the SMS contents are shown in Table 1 below.

**Table 1:** 

CORE MESSAGES
Control of your blood glucose level require you to eat good food, do exercise and regularly use your pills/insulin as prescribed. Your nurse, dietician and doctor can assist you.
You are the most important member of your healthcare team because you are the one who manage your diabetes day by day.
It is important to know your blood glucose level overtime because you do not want your blood sugar to get too high.
Do you know if your sugar is normal or close to normal, you have less chances of developing heart problems, stroke, eye problems and kidney problems? The answer is YES!!!
**HEALTHY EATING**
Eating healthy diet is an important aspect of your diabetes management. It will help in controlling your blood glucose level.
Ensure you make a diabetes meal plan with the help of your nurse.
Choose food such as fruits, vegetables, whole grains, bread, cereals, low-fat or skimmed milk and cheese.
Water is the best drink. Choose water rather than juice, regular soda, Twizza drink or coke
Avoid using too much margarine, butter, mayo or salad dressing
**STRESS AND MOOD MANAGEMENT MESSAGE**
Hello [name]. Too much stress can increase your blood sugar. Make sure you have fun and do something you enjoy today. This will help you reduce stress and improve how you feel.
Are you feeling down? If yes, ask help from a friend, family member, clergy, counsellor or your nurse today.
**REMINDERS**
Have you taken your pills today?
Hello [Name]. Did you check your sugar level today or recently?
Hi [Name]. Tomorrow is your next appointment visit to your nurse, do not forget. Going for your appointment help you and your nurse/doctor manage your diabetes better.
**FOOT CARE**
Check your feet regularly for blisters, red spots or swelling
Looking after your feet will help you prevent foot problems in the future
**EXERCISE**
Set a goal to become more active most days of the week. Start slow by taking 10 min' walk, three times a day.
Stay at or get healthy weight using your planned diets and doing more exercise
**SMOKING AND ALCOHOL**
Avoid taking alcohol in order to better control your diabetes level
Hi [Name]. Good management of your diabetes includes not smoking. Talk to your nurse about how they can help.
**GENERAL**
Brush your teeth daily and floss to keep your mouth, teeth and gums healthy
Report any changes you observe in your health to your nurse/doctor.

## 13 Data analysis

At baseline, relevant summary statistics was used to assess for similarity in the baseline information across the two study groups. Afterwards, all imbalances were adjusted for. The treatment evaluations will be performed based on the principle of intention-to-treat (ITT). Continuous variables will be summarised as numbers of observed values, means, standard deviation, median, minimum and maximum. Categorical variables will be described as frequency and percentage. Chi-square and Fisher’s exact test will be used to assess the difference between groups for categorical baseline variables. For continuous variables, analysis of variance will be used to assess the difference in the baseline characteristics of the study participants between the intervention and control group. The effect of the intervention on the primary outcome between the two groups and at the two periods will be assessed using the random effects mixed-model analysis. Linear regression will be used to assess the effect of the intervention on secondary outcome measures between the two groups and two periods. All statistical tests will be two-sided at 5% significance level. The Statistical Package for Social Sciences (SPSS) version 22 was be used for data analysis.

## 14 Ethics and dissemination

The ethical approval for this study was obtained from the University of Fort Hare Research Ethics Committee. After this, approval was sought from the Eastern Cape Departments of health. On receiving this, the approval from all the included sub-districts was also obtained. Also, approval was obtained from the clinic managers. Lastly, verbal and written informed consent was obtained from the participants before the commencement of the study, after due explanation of the research purpose and aims. Rights to anonymity and confidentiality will be ensured throughout the study and participants consented to referral to further care in case of detection of abnormal findings.

Data obtained will be published in peer reviewed journals and presented at conferences after due communication with the patients, health district, relevant stakeholders and the general public.

## 15 Discussion

This paper presents the protocol for a two-arm, parallel RCT aimed at assessing the effectiveness, acceptability and feasibility of mHealth technology in promoting treatment outcomes among diabetic patients with uncontrolled glycaemic status in the low-resource settings of the Eastern Cape Province of South Africa. The specific mHealth technology being evaluated is the mobile phone SMS and the particular treatment outcomes of interest are adherence to medication and lifestyle regimen, diabetes knowledge, health-related quality of life, clinic attendance and clinical outcomes such as change in mean blood glucose, blood pressure and weight. It has been documented that the use of innovative technologies, including the SMS is a measure of promoting and improving health, even at a low-cost [[27], [28], [29]]. The use of the mobile phone SMS will assist in overcoming barriers in information dissemination regarding diabetes, its management and for enhancing the required behavioural change, if effective.

Even though there are documented studies on the use of mobile phone SMS in promoting adherence and glycaemic control in many developed countries and few developing countries [[25], [26], [27], [28], [29]], there are no such studies on diabetes in South Africa. Although its use for other chronic health conditions such as hypertension has been documented, it is however important to evaluate its effectiveness with regards to diabetes on its own, considering the complexity of diabetes management and expected lifestyle and behavioural modifications.

This intervention uses a multi-centre approach which is rarely in use in previous studies of this nature, especially in developing countries and this approach is sometimes preferable [24]. Also, the use of the multi-centre approach will assist in prompt recruitment of the participants as there are hardly any record of patients in place in majority of the clinics, so we rely only on whichever diabetic patient visits the clinic during the recruitment period. Nonetheless, this study will be limited to few centres due to time and financial restraints. Also, to avoid bias, randomisation will only take place after identification of potential, eligible participants.

Likewise, we focused on the low-resource areas and we chose to evaluate this intervention at the primary healthcare level where human power, and resources are limited, yet, where majority of the chronic patients are managed. This intervention is not considered tedious as only a minimal technological skill is required. The short messages will be sent using an internet-based bulk SMS platform with options to show the status of the message as either delivered, read or not delivered. This study differs in that it is not conducted in urban areas as usually done in previous studies and it is targeted at individuals with little level of education who are usually found in these areas.

It is worthy of note that the HBA1c which is considered the gold standard will not be used as a yardstick for measuring glycaemic control in this study. This is owing to the fact that HBA1c is rarely conducted at the prescribed interval for the patients with uncontrolled diabetes in this settings, despite being emphasised in the diabetes management guideline [32]. The unpublished data gathered during the preliminary studies shows that less than a quarter of these patients has had their HBA1c level evaluated in the last six months. As such, we rely on the most feasible and practical measure at this level of healthcare which is the morning random blood sugar. This test, even though not considered a gold standard is seen as an easy measure and is the more feasible option sometimes used in making clinical judgement at this level of healthcare in this setting. Studies have already correlated this measure with the HBA1c and supports that it be used with caution in resource-limited settings where access to HBA1c test is limited. However, in order to give room to little changes, the average of the blood sugar reading at the point of data collection and the two previous readings in the last two months will be used in this study, since the majority of the patients visit the clinic every month and will likely get this test done at each visit.

The findings of this study will assist in determining the effectiveness of this innovative measure even in the least opportuned level of health care system where many of these patients are being managed.

## Trial registration

This trial was registered in the Pan African Clinical Trial Registry, trial number: PACTR201810599931422.

## Consent for publication

Both authors approved the submission of this final draft towards publication in a peer reviewed journal.

## Availability of data and materials

Data from this study will be made available on request.

## Conflicts of interest

The authors declare no conflict of interest.

## Authors’ contributions

EOO and DTG conceptualised and designed the study. EOO drafted the paper and DTG made intellectual contributions to the draft.

## Funding

EOO received doctoral study grant in her third year of study from the National Research Foundation, South Africa.

## References

[1] World Health Organization (2016). Global Report on Diabetes, Geneva, Switzerland.

[2] International Diabetes Federation (2013). IDF Diabetes Atlas. http://www.idf.org/diabetesatlas.

[3] World Health Organization Diabetes Mellitus. Factsheet. http://www.who.int/mediacentre/factsheets/fs138/en/.

[4] Anjana R.M., Pradeepa R., Deepa M., Datta M., Sudha V. (2011 Sep 30). Prevalence of diabetes and prediabetes (impaired fasting glucose and/or impaired glucose tolerance) in urban and rural India: phase I results of the Indian Council of Medical Research–India DIABetes (ICMR–INDIAB) study. Diabetologia.

[5] Dasappa H., Fathima F., Prabhakar R., Sarin S. (2015). Prevalence of diabetes and pre-diabetes and assessments of their risk factors in urban slums of Bangalore. Journal of Family Medicine and Primary Care [Internet]. Medknow.

[6] Hwang C.K., Han P.V., Zabetian A., Ali M.K., Venkat Narayan K.M. (2012 Jun). Rural diabetes prevalence quintuples over twenty-five years in low- and middle-income countries: a systematic review and meta-analysis. Diabetes Res. Clin. Pract..

[7] Shen J., Kondal D., Rubinstein A., Irazola V., Gutierrez L., Miranda J.J. (2016 Mar). A multiethnic study of pre-diabetes and diabetes in LMIC. Glob. Heart.

[8] International Diabetes Federation (2015). Diabetes in South Africa, South Africa. http://www.idf.org/membership/afr/south-africa.

[9] Guariguata L., Whiting D.R., Hambleton I., Beagley J., Linnenkamp U., Shaw J.E. (2014 Feb). Global estimates of diabetes prevalence for 2013 and projections for 2035. Diabetes Res. Clin. Pract..

[10] Pillay-van Wyk V., Dorrington R.E., Bradshaw D. (2017 Feb 27). Rapidly changing mortality profiles in South Africa in its nine provinces. S. Afr. Med. J..

[11] Adisa R., Fakeye T. (2014 Mar 7). Treatment non-adherence among patients with poorly controlled type 2 diabetes in ambulatory care settings in southwestern Nigeria. African Health Sciences [Internet]. Afr. J. Online AJOL.

[12] Bagonza J., Rutebemberwa E., Bazeyo W. (2015 Apr 19). Adherence to anti diabetic medication among patients with diabetes in eastern Uganda; a cross sectional study. BMC Health Serv. Res..

[13] Shilubane N.H. (2010 Jun 6). Factors contributing to poor glycaemic control in diabetic patients at Mopani District. Curationis [Internet]. AOSIS.

[14] Booysen B.L., Schlemmer A.C. (2015 Mar 16). Reasons for diabetes patients attending Bishop Lavis Community Health Centre being non-adherent to diabetes care. S. Afr. Fam. Pract..

[15] Kagee A. (2004 Nov). Treatment adherence in South African primary health care. S. Afr. Fam. Pract..

[16] Liang X., Wang Q., Yang X., Cao J., Chen J., Mo X. (2011 Mar 10). Effect of mobile phone intervention for diabetes on glycaemic control: a meta-analysis. Diabet. Med..

[17] Wu Y., Yao X., Vespasiani G., Nicolucci A., Dong Y., Kwong J. (2017 Mar 14). Mobile app-based interventions to support diabetes self-management: a systematic review of randomized controlled trials to identify functions associated with glycemic efficacy. JMIR mHealth uHealth.

[18] Dobson R., Whittaker R., Pfaeffli Dale L., Maddison R. (2017 Jan). The effectiveness of text message-based self-management interventions for poorly-controlled diabetes. Syst. Rev. Digit. Health.

[19] Arora S., Peters A.L., Burner E., Lam C.N., Menchine M. (2014 Jun). Trial to examine text message–based mHealth in emergency department patients with diabetes (TExT-MED): a randomized controlled trial. Ann. Emerg. Med..

[20] Adeniyi O.V., Yogeswaran P., Longo-Mbenza B., Goon D.T., Reboldi G. (2016 Mar 1). Uncontrolled hypertension and its determinants in patients with concomitant type 2 diabetes mellitus (T2DM) in rural South Africa.

[21] Déglise C., Suggs L.S., Odermatt P. (2012 Jan 12). Short message service (SMS) applications for disease prevention in developing countries. J. Med. Internet Res..

[22] Jemberu S. (2013). Mobile Health Application in Ethiopia: Existing Initiatives and Practices.

[23] Jha P., Ramasundarahettige C., Landsman V., Rostron B., Thun M., Anderson R.N. (2013 Jan 24). 21st-Century hazards of smoking and benefits of cessation in the United States. New england journal of medicine [internet]. N. Engl. J. Med..

[24] Islam S.M.S., Lechner A., Ferrari U., Froeschl G., Alam D.S., Holle R. (2014 Nov 26). Mobile phone intervention for increasing adherence to treatment for type 2 diabetes in an urban area of Bangladesh: protocol for a randomized controlled trial. BMC Health Serv. Res..

[25] Lester R.T., Gelmon L., Plummer F.A. (2006 Nov). Cell phones: tightening the communication gap in resource-limited antiretroviral programmes? AIDS [Internet]. Ovid Technologies (Wolters Kluwer Health).

[26] Sinha S.R., Barry M. (2011 Sep). Health technologies and innovation in the global health arena. New england journal of medicine [internet]. N. Engl. J. Med..

[27] Goodarzi M., Ebrahimzadeh I., Rabi A., Saedipoor B., Jafarabadi M. (2012). Impact of distance education via mobile phone text messaging on knowledge, attitude, practice and self-efficacy of patients with type 2 diabetes mellitus in Iran. J. Diabetes Metab. Disord..

[28] Quinn C., Shardell M., Terrin M., Barr E., Ballew S., Gruber-Baldini A. (2011). Cluster-randomized trial of a mobile phone personalized behavioral intervention for blood glucose control. Diabetes Care.

[29] Islam S.M., Niessen L.W., Ferrari U., Ali L., Seissler J., Lechner A. (2015 Jul 23). Effects of Mobile Phone SMS to Improve Glycemic Control Among Patients with Type 2 Diabetes in Bangladesh: A Prospective, Parallel-Group, Randomized Controlled Trial. Diabetes Care.

[30] Chan A.-W., Tetzlaff J.M., Altman D.G., Laupacis A., Gøtzsche P.C., Krleža-Jerić K. (2013 Feb 5). SPIRIT 2013 statement: defining standard protocol Items for clinical trials. Ann. Intern. Med..

[31] Eysenbach G. (2011 Dec 31). CONSORT-EHEALTH: improving and standardizing evaluation reports of web-based and mobile health interventions. J. Med. Internet Res..

[32] Amod A., Motala A., Levitt N., Berg J., Young M. (2017). Type 2 diabetes guideline. J. Endocrinol. Metabol. Diabetes S. Afr..

[33] Govender R.D., Gathiram P., Panajatovic M. (2017). Poor control and management of type 2 diabetes mellitus at an under-resourced South African Hospital: is it a case of clinical inertia?. S. Afr. Fam. Pract..

[34] Daramola O.F. (2012). Assessing the Validity of Random Blood Glucose Testing for Monitoring Glycemic Control and Predicting Hba1c Values in Type 2 Diabetics at Karl Bremer Hospital. Thesis.

[35] Reidpath D.D., Jahan N.K., Mohan D., Allotey P. (2016 Aug 9). Single, community-based blood glucose readings may be a viable alternative for community surveillance of HbA1c and poor glycaemic control in people with known diabetes in resource-poor settings. Glob. Health Action.

[36] Makris K., Spanou L., Rambaouni-Antoneli A., Koniari K., Drakopoulos I., Rizos D. (2008 Feb).

[37] Rohlfing C.L., Wiedmeyer H.-M., Little R.R., England J.D., Tennill A., Goldstein D.E. (2002 Feb 1). Defining the relationship between plasma glucose and HbA1c: analysis of glucose profiles and HbA1c in the diabetes control and complications trial. Diabetes care [internet]. Am. Diabetes Assoc..

[38] Rosediani M., Azidah A.K., Mafauzy M. (2006). Correlation between fasting plasma glucose, post prandial glucose and glycated haemoglobin and fructosamine. Med. J. Malays..

[39] Fitzgerald J.T., Funnell M.M., Anderson R.M., Nwankwo R., Stansfield R.B., Piatt G.A. (2016 Jan 14). Validation of the revised brief diabetes knowledge test (DKT2). Diabetes Educat..

[40] Devlin N.J., Brooks R. (2017 Feb 13). EQ-5D and the EuroQol group: past, present and future. Appl. Health Econ. Health Policy.

[41] Schmitt A., Reimer A., Hermanns N., Huber J., Ehrmann D., Schall S., Wang X. (2016 Mar 3). Assessing diabetes self-management with the diabetes self-management questionnaire (DSMQ) can help analyse behavioural problems related to reduced glycaemic control.

[42] Schmitt A., Gahr A., Hermanns N., Kulzer B., Huber J., Haak T. (2013). The Diabetes Self-Management Questionnaire (DSMQ): development and evaluation of an instrument to assess diabetes self-care activities associated with glycaemic control. Health Qual. Life Outcomes.

